# Current Understanding on Why Ovarian Cancer Is Resistant to Immune Checkpoint Inhibitors

**DOI:** 10.3390/ijms241310859

**Published:** 2023-06-29

**Authors:** Anna Pawłowska, Anna Rekowska, Weronika Kuryło, Anna Pańczyszyn, Jan Kotarski, Iwona Wertel

**Affiliations:** 1 Independent Laboratory of Cancer Diagnostics and Immunology, Department of Oncological Gynaecology and Gynaecology, Faculty of Medicine, Medical University of Lublin, Chodźki 1, 20-093 Lublin, Poland; anna.pawlowska@umlub.pl (A.P.); iwonawertel@umlub.pl (I.W.); 2 Students’ Scientific Association, Independent Laboratory of Cancer Diagnostics and Immunology, Medical University of Lublin, Chodźki 1, 20-093 Lublin, Poland; arekowska@icloud.com (A.R.); weronika.kurylo@vp.pl (W.K.); 3 Institute of Medical Sciences, Department of Biology and Genetics, Faculty of Medicine, University of Opole, Oleska 48, 45-052 Opole, Poland; plo.anna@wp.pl

**Keywords:** ovarian cancer, immune checkpoints, PD-1/PD-L1, TIGIT, immunotherapy, resistance, microRNA

## Abstract

The standard treatment of ovarian cancer (OC) patients, including debulking surgery and first-line chemotherapy, is unsatisfactory because of recurrent episodes in the majority (~70%) of patients with advanced OC. Clinical trials have shown only a modest (10–15%) response of OC individuals to treatment based on immune checkpoint inhibitors (ICIs). The resistance of OC to therapy is caused by various factors, including OC heterogeneity, low density of tumor-infiltrating lymphocytes (TILs), non-cellular and cellular interactions in the tumor microenvironment (TME), as well as a network of microRNA regulating immune checkpoint pathways. Moreover, ICIs are the most efficient in tumors that are marked by high microsatellite instability and high tumor mutation burden, which is rare among OC patients. The great challenge in ICI implementation is connected with distinguishing hyper-, pseudo-, and real progression of the disease. The understanding of the immunological, molecular, and genetic mechanisms of OC resistance is crucial to selecting the group of OC individuals in whom personalized treatment would be beneficial. In this review, we summarize current knowledge about the selected factors inducing OC resistance and discuss the future directions of ICI-based immunotherapy development for OC patients.

## 1. Heterogeneity and Prognosis of Ovarian Cancer (OC)

Despite the progress made in the treatment of solid malignancies, ovarian cancer (OC) continues to be the most lethal gynecological cancer. In 2020, OC was diagnosed in 313,959 women, and as many as 207,252 patients died because of the disease. According to the prediction of the World Health Organization (WHO), in 2025, the number of newly diagnosed OC patients will total 823,315 [1].

The prognosis for patients with OC is poor because the disease symptoms are unspecific at early stages (stages I–II of the International Federation of Gynecology and Obstetrics—FIGO). Thus, OC in <70% of all cases is diagnosed at FIGO stages III and IV when the tumor has already spread to distant organs. Ovarian cancer at FIGO stages I and II is accounted as curable, and the five-year survival rates total 90% and 70%, respectively. In comparison, at the advanced stages of OC, the five-year survival rate drops below 30% [2,3,4,5].

Moreover, heterogeneity has an impact on the diagnosis and high mortality rate. Based on different morphology, the WHO classifies OC cases into several subtypes, including transitional-cell Brenner tumors, serous, mucinous, clear-cell, endometrioid carcinomas, and mixed and undifferentiated types. However, this classification is insufficient because it does not take into account the molecular background, the prognosis, or the etiology of the disease [6,7]. Histologically, high-grade serous ovarian carcinoma (HGSOC) accounts for approximately 80% of epithelial OC cases. Up to 75% of HGSOC cases are diagnosed at the advanced stages (FIGO stages III and IV) of the disease [8].

Kurman and Shih classified OC into two types, allowing for the dualistic model of carcinogenesis. Their classification includes genetic mutations, molecular biology, and histopathological background. Type I includes endometrioid, low-grade serous, seromucinous, mucinous, malignant Brenner tumor, and clear cell carcinoma. These are genetically stable carcinomas that are mostly diagnosed at early stages (FIGO stages I and II). The prognosis for patients with type I OC is favorable (with a mortality rate of 10% and a slow rate of disease progression) [9,10].

Unfortunately, the prognosis for patients with type II tumors is poor. The majority of cases (~75%) are diagnosed at advanced FIGO stages. Type II OC includes carcinosarcomas, undifferentiated carcinomas, and high-grade serous carcinomas. In contrast to type I, they are highly aggressive and develop rapidly. Ascites are a frequently occurring symptom. Type I and type II OC cases also differ in somatic mutations. In type I, the most frequent mutations include phosphatase and tensin homolog deleted on chromosome 10 (PTEN), extracellular signal-regulated kinase (ERK), AT-rich interactive domain-containing protein 1A (ARID1A), B-Raf proto-oncogene, serine/threonine kinase (BRAF), mitogen-activated protein (MAP), phosphatidylinositol 4,5-bisphosphate 3-kinase catalytic subunit α (PIK3CA), and Kirsten rat sarcoma viral oncogene homolog (KRAS). In type II OC, mutations in *RB1* (gene encoding retinoblastoma protein), *TP53* (Tumor protein P53), *FOXM1* (forkhead box M1), genes encoding cyclin E, and Notch3 are the most frequent [9,10].

## 2. Treatment of Ovarian Cancer

The standard treatment of OC patients includes debulking surgery and carboplatin and paclitaxel chemotherapy as part of first-line treatment [11,12,13]. However, the majority of patients (~70%) suffering from advanced OC experience recurrences. As a result, the disease becomes non-sensitive to platinum-based chemotherapy [3,14]. The efficiency of second-line therapy that includes gemcitabine or pegylated liposomal doxorubicin (PLD) is poor. Thus, it is necessary to develop other treatment strategies with a view to improving long-term clinical outcomes [13,15].

Clinical trials have demonstrated that targeted molecular drugs improve the outcomes of patients with advanced OC [13]. The Food and Drug Administration (FDA) approved two biological medical preparations, namely bevacizumab (vascular endothelial growth factor inhibitor; VEGFi) in 2018 and olaparib (poly(ADP-ribose) polymerase inhibitor; PARPi) in 2014. The combination of these drugs was approved by the FDA in 2020 for breast cancer gene (*BRCA*)-mutated OC [16].

Despite the progress made in OC treatment, the prognosis for patients remains poor. This is related to the lack of screening biomarkers in clinical practice and the heterogeneity of the disease. The diagnosis of OC is primarily based on the presence of cancer antigen 125 (CA-125) in serum, diagnostic imaging, and laparoscopy. However, these approaches are insufficient to detect the disease at early stages [3,14,17,18,19,20].

Considering the unsatisfactory efficacy of standard therapies, interactions in the tumor microenvironment (TME) seem to be potential targets in OC treatment. The signals derived from TME manipulate the activity and functions of immune cells and lead to immune evasion by cancer cells via various mechanisms, including immune checkpoints (ICPs), such as programmed cell death pathways and cytotoxic T-lymphocyte-associated antigen 4 (CTLA-4) [5,21,22,23,24,25,26,27]. It should be stressed that the activity of these molecules prevents autoimmunity in normal conditions, but their upregulation leads to the suppression of immune response [28]. Targeting immune checkpoints and their blockade by monoclonal antibodies (mAbs) lead to restoring the sensitization of the immune system to cancer cells [29].

Programmed cell death receptor 1 (PD-1) belongs to the CD27 immunoglobulin superfamily and is encoded by the programmed cell death 1 (*PDCD1*) gene (chromosome 2) [30]. It is expressed on CD4^+^ and CD8^+^ T cells and antigen-presenting cells (APCs), including B cells, dendritic cells (DCs), and monocytes/macrophages (MO/MA). The PD-1 receptor (CD279) ligands include programmed death-ligand 1 (PD-L1; CD274, B7-H1) and programmed death-ligand 2 (PD-L2; CD273, B7-DC). They are both expressed on APCs and tumor cells. The binding of the ligand (PD-L1 or PD-L2) on a tumor cell with PD-1 receptor on a T cell leads to the exhaustion of the T cell and the inhibition of its effector activity. Moreover, the interaction results in the enhanced secretion of proinflammatory cytokines, including interferon γ (IFN-γ), tumor necrosis factor α (TNF-α), and interleukin 2 (IL-2) [5,23,31,32]. Consequently, the ability of T cells to eliminate cancer cells is decreased, and they are able to escape immune surveillance. The PD-L1 expression in tumor cells, which is upregulated by chemopreventive factors, results in a decreased T cell activity targeting cancer cells and in promoting the tumor cells’ evasion of surveillance by immune cells. This suggests a relationship between immune resistance and chemotherapy in OC patients [33,34].

Another co-inhibitory molecule that plays a crucial role in OC progression and tumorigenesis is CTLA-4 [35]. It is a membrane protein expressed by activated T cells, constitutively regulatory T cells (Tregs), and is considered to be homologous to CD28, which is involved in the second step of T cell activation after the binding of an antigen and T cell receptor (TCR). Notably, CTLA-4 and CD28 share the same ligands, CD80 (B7-1) and CD86 (B7-2). However, the affinity of CTLA-4 for each ligand is 500–2500 times higher in comparison with that of CD28. In contrast to CD28 activity, the result of CTLA-4 and CD80/86 binding is the suppression of immune response [21]. The TCR signaling is suppressed, and T cells’ activity is inhibited by interactions between CTLA-4 expressed T cells and its ligands expressed on APCs in lymph nodes. In consequence, the anti-tumor immune response is suppressed by inhibiting the effector activity of T cells at an early stage of T cell activation [31,36].

The role of both ICPs, i.e., the PD-1/PD-L1/PD-L2 pathway and CTLA-4, in inhibiting anti-tumor response are similar. However, CTLA-4 regulates the immune response at an early stage in lymph nodes, whereas the PD-1/PD-L1/PD-L2 pathway regulates anticancer immune response at later stages in peripheral tissues [31].

Immunotherapies based on ICPs targeted against PD-1 and its ligands (PD-L1, PD-L2), as well as CTLA-4, turned out to be game-changers in the treatment of various malignancy types [37]. These immune checkpoint inhibitors (ICIs) improve the overall survival (OS) rate in malignancies with inflamed TME, non-small-cell lung cancer (NSCLC) [38,39,40], melanoma [41,42], renal cancer [43], head and neck squamous cell carcinoma (HNSCC) [44], and urothelial carcinoma [45].

**Figure 1:**
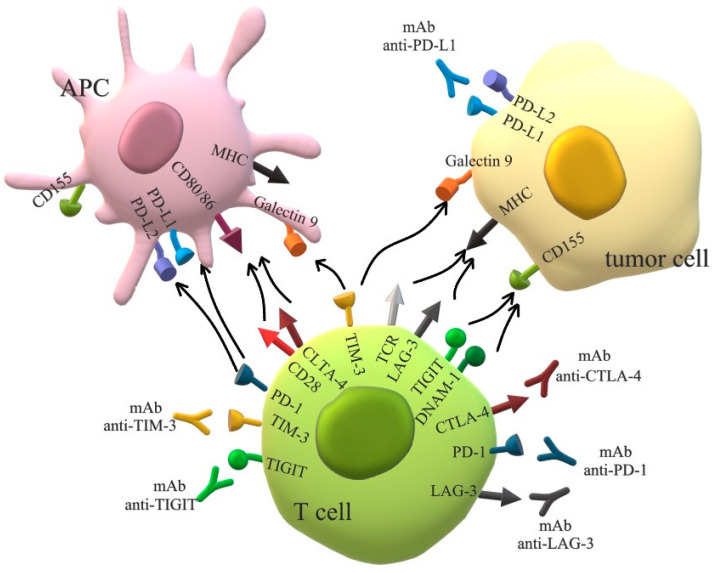
The modes of action of selected ICPs and ICIs.

Despite the fact that ICI immunotherapy is not as effective as in other solid malignancies (with the response rate to monotherapy in OC patients totaling 10–15%) [46,47], the clinical trials that are currently being conducted determine its impact in monotherapy and/or in combination with other agents, such as biological drugs or standard therapy, to improve OC patients’ outcomes [48,49]. The modes of action of selected ICPs and ICIs are presented in Figure 1.

It is noteworthy that OC, similar to breast cancer, is a hormone-dependent tumor in which steroid hormones (estrogen, progesterone) and their receptors (estrogen receptor (ER) and progesterone receptor (PR)) influence the disease progression. In addition to the fact that ERs are potential targets in OC treatment, their modulators and enzymes are also involved in estrogen synthesis [50,51].

Aromatase is an enzyme (synthetase) that is crucial in estrogen synthesis and its circulation. Its inhibitors, such as exemestane, letrozole, and anastrozole, inhibit the shift from androgen to estrogen, downregulating the circulating estrogen level [52,53].

Hormone therapy, including anti-estrogen treatment (tamoxifen) and aromatase [54] inhibitors, is efficient for ER-positive OC patients with recurrence episodes or advanced stages of the disease. Additionally, it appears as a treatment characterized by low toxicity. However, due to the heterogeneity of OC, studies conducted on small samples, variable expression of hormones on OC cells, and the lack of biomarkers, the therapeutic value of this kind of OC treatment is inconclusive. It should be highlighted that the network of interrelationships of hormonal modulation is complex and concerns not only estrogen, progesterone, their receptors, and aromatase but also signaling cascades, including the Janus kinase/signal transducers and activators of transcription (JAK-STAT), mitogen-activated protein kinases (MAPK), Src, and receptor tyrosine kinase [50,51,55,56]. Further multicenter clinical studies are necessary to confirm the efficacy of the treatment. However, the hormonal modulation is not the subject matter of this paper.

## 3. Clinical Trials in Ovarian Cancer

Currently, there are nine ICIs approved by the FDA for use in cancer treatment. These are divided into three groups: anti-PD-1/PD-L1 mAbs (pembrolizumab, nivolumab, cemiplimab, atezolizumab, durvalumab, avelumab), anti-CTLA-4 mAbs (ipilimumab, tremelimumab), and anti-LAG-3 mAbs (relatlimab) [54].

To date, ICIs have been approved for various types of malignancies. Pembrolizumab, nivolumab, and cemiplimab are anti-PD-1 mAbs approved for the treatment of melanoma, NSCLC, malignant mesothelioma, HNSCC, classical Hodgkin Lymphoma (cHL), primary mediastinal large B-cell lymphoma (PMLBCL), urothelial cancer, microsatellite instability-high (MSI-H) or mismatch repair (dMMR) deficient colorectal cancer (CRC), hepatocellular carcinoma (HCC), renal cell carcinoma (RCC), esophageal cancer, gastric cancer, gastroesophageal junction cancer, cervical cancer, endometrial cancer, high tumor mutational burden (TMB-H) cancers, cutaneous squamous cell carcinoma (cSCC), and triple-negative breast cancer [57,58,59].

Anti-PD-L1 mAbs, i.e., atezolizumab, durvalumab, and avelumab, are approved for the treatment of melanoma, NSCLC, small cell lung cancer (SCLC), HCC, urothelial carcinoma, biliary tract cancers (BTC), and metastatic Merkel cell carcinoma (MCC) [60,61,62].

CTLA-4 blocking mAbs are approved for the treatment of HCC, NSCLC, melanoma, renal cell carcinoma, mesothelioma, CRC, and cutaneous squamous cell carcinoma [55,56]. However, the incidence of immune-related adverse events (irAEs) is higher in cancer patients treated with ipilimumab as a single agent (86%) compared with the treatment with nivolumab alone (78%) and with combined therapy using both agents (95%) [57,58,63,64,65,66].

Relatlimab is a LAG-3 blocking mAb approved in combination with nivolumab for the treatment of unresectable or metastatic melanoma [67].

To date, OC has been one of the few tumors for which ICI-based treatment has not been approved, either as part of combined therapy or as monotherapy [68]. According to the European Society For Medical Oncology (ESMO) guidelines, the use of ICIs is not applicable in OC [69]. However, the National Comprehensive Cancer Network (NCCN) Clinical Practice Guidelines resonate and recommend using ICIs in certain cases, i.e., dostarlimab-gxly for recurrent or advanced dMMR or MSI-H tumors and pembrolizumab for MSI-H or dMMR solid tumors, as well as for patients with TMB-H tumors with ≥10 mutations/megabase [70].

The combination of immunotherapies based on ICPs, VEGFi, and PARPi, and the development of biomarkers of ICI efficiency appears to provide a promising strategy for OC treatment [68]. Researchers have recently focused on folate receptor alpha (FRα) which was found to be overexpressed in 70–90% of OC cases and, therefore, became a promising target for anticancer drug development [71]. In November 2022, a novel FRα-directed antibody and microtubule inhibitor conjugate (mirvetuximab soravtansine) was granted accelerated approval for use in FRα positive, platinum-resistant epithelial ovarian, fallopian tube, and peritoneal cancer [72].

According to ClinicalTrials.gov, there are 125 ongoing clinical trials concerning anti-PD-1 agents, 111 studies of anti-PD-L1 mAbs, and 26 research projects concerning anti-CTLA-4 mAbs in the treatment of OC [73]. Most of them are combined with other drugs and/or biological agents. The advanced-phase clinical trials (phases 3 and 4) concerning ICIs in OC treatment are presented in Table 1.

It should be highlighted that there are also clinical trials that concern other ICIs in the treatment of OC, including lymphocyte activation gene 3 (LAG-3), i.e., relatlimab, INCAGN02385, and T cell immunoglobulin and ITIM domain (TIGIT) inhibitors (COM902, etigilimab). The current research examines the use of bispecific mAbs in OC treatment, such as XmAb^®^22841 (anti-CTLA-4 and anti-LAG-3) [89] and tebotelimab (anti-PD-1 and anti-LAG-3) [90]. However, these studies are in the early phases (phases 1–2). Selected early-phase clinical trials concerning ICIs in OC treatment are presented in Table 2.

According to ClinicalTrials.gov (accessed on 2 June 2023), there are 217 ongoing clinical trials focusing on ICIs in the treatment of OC [95]. These studies concern ICIs in monotherapy, as well as in combination with other agents, such as biological drugs and standard therapy. Selected trials are summarized in Table 3.

## 4. Mechanisms of Immunotherapy Resistance in Ovarian Cancer

Despite the successful use of ICIs in the treatment of other solid malignancies, their efficacy in OC therapy is insufficient. Thus, the understanding of biological, molecular, and genetic mechanisms of immunotherapy resistance in OC patients plays a crucial role in developing response biomarkers. It would be helpful in selecting a group of OC individuals for whom this kind of treatment would be beneficial, as well as projecting efficient targeted (immuno)therapies. It should be highlighted that the majority of clinical trials concerning the use of ICIs in OC treatment focus on heavily pretreated individuals, including patients with the disease recurrence. Drakes et al. [96] have shown higher PD-1 expression on T cells and PD-L1 expression on tumor cells at early OC stages in comparison with the advanced stages of the disease. Thus, clinical trials using ICIs in the first line of OC treatment seem to be crucial in the pursuit of implementing it in clinical practice. To date, numerous factors that determine the response of OC patients to ICI-based immunotherapy have been identified, including the heterogeneity of TME, as well as the molecular and genetic background.

### 4.1. Significance of Tumor Infiltrating Lymphocytes (TILs)

There are several variables that influence the success of ICIs in OC treatment, including their interactions with and influence on tumor-infiltrating lymphocytes (TILs). The cells belonging to this subset express multiple molecules, including immune checkpoints such as T cell immunoglobulin, mucin domain-containing protein 3 (Tim-3), LAG-3, CTLA-4, and PD-1 [48]. It should be emphasized that the response of cancer patients to ICIs depends on TME heterogeneity, including inflamed (hot) tumors with high infiltration of T cells and low immune-reactive tumors, i.e., non-inflamed (cold) tumors with low infiltration by T cells, ‘immune-excluded’ tumors where TILs are observed only in stromal space, or ‘immune desert’ tumors with no TILs present in TME.

**Figure 2:**
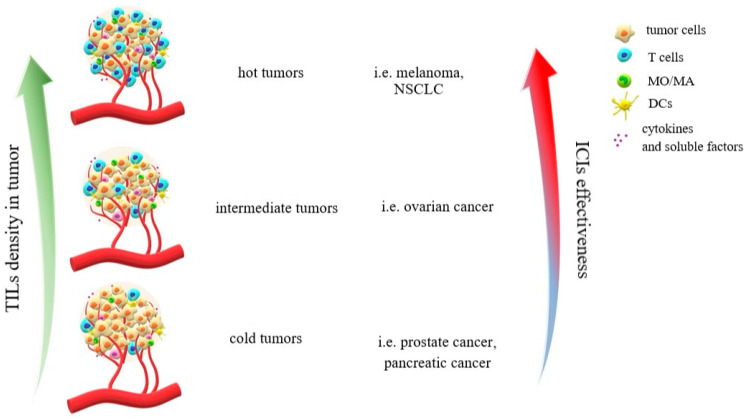
The main features of hot, intermediate, and cold tumors.

The inflammatory tumors display an effective response to the immunomodulatory compounds and have a favorable prognosis. However, OC is considered a cold or warm tumor with low to intermediate infiltration by T cells [30,36,97,98,99]. Such malignancies as prostate, breast, pancreatic, and colorectal cancers are also regarded as cold tumors [30]. Cancer cells from non-inflamed tumors display only a modest level of neoantigens and have a low mutational burden and a negative/low PD-L1 expression. As a result, effector cells of the immune system are not able to distinguish them from normal cells, thereby prompting cancer cells to evade the immune system [29,30]. Thus, the presence of TILs and PD-1 expression are considered to be positive prognostic factors [8,100,101,102]. The response to ICIs is higher in PD-L1 positive tumors, but the high PD-L1 level is related to poor prognosis [30]. The functions of TILs are inhibited by immunosuppressive TME, which leads to an ineffective elimination of tumor cells [27]. The main features of hot, intermediate, and cold tumors are presented in Figure 2.

### 4.2. Dual Role of Tumor-Associated Macrophages (TAMs)

Tumor-associated macrophages (TAMs) comprise the main subset of immune system cells in OC TME and arise either from bone marrow monocytes or tissue-resident macrophages [103,104]. It should be stressed that TAMs have a dual nature depending on their phenotypes. There are two phenotypes of TAMs: the first one is the tumor-suppressive M1 type, and the second one is the tumor-promoting M2 type. The M2 macrophages, in addition to producing vascular endothelial growth factor (VEGF), epidermal growth factor (EGF), transforming growth factor β (TGF-β), and hepatocyte growth factor (HGF), also enhance the maturation of regulatory T cells via TGF-β and the infiltration of the tumor by M2 TAMs via chemokine (C-C motif) ligand 2 (CCL2) and colony-stimulating factor 1 (CSF-1). Moreover, TAMs secrete IL-6 and IL-10 that upregulate B7-H4 and, consequently, block T cell functions [103,105,106].

M2-like macrophages are a population of immune system cells playing a key role in the creation of immunosuppressive TME in metastatic OC. They are involved in cytokine and chemokine signaling, such as IL-10, C-C motif chemokine 22 (CCL22), IL-4 (components), and IL-13 (components) signaling pathways, leading to T cell exhaustion. It has been proven that M2-like TAMs suppress immune responses in HGSOCs [107]. Yin et al. [108] have shown that, in the peritoneal fluid of OC patients during the disease progression, TAMs are polarized into an M2-like population that leads to the enhancement of OC cell migration and proliferation [108]. Mei Song et al. [106] have revealed that ubiquitin-protein ligase E3 component n-recognin 5 (*UBR5*), a gene that is frequently overexpressed in OC, plays an important role in the creation of immunosuppressive TME. The authors have further demonstrated that *UBR5* deficiency impairs TAMs recruitment. Moreover, mice with an ovarian tumor subjected to treatment targeting tumor-derived *UBR5*, concurrently with anti-PD-1 mAbs, responded to the therapy, whereas mice treated only with anti-PD-1 agents did not [106].

Another protein expressed by TAMs is the transmembrane protein triggering receptor on myeloid cells 2 (TREM2). Notably, TREM2 causes T cell exhaustion and anti-PD-1 resistance. Binnewies et al. [109] have reported that TME in which TAMs express TREM2 displays immunosuppressive properties, which results in maintaining resistance to anti-PD-1 treatment [109]. The authors have found that the level of TREM2^+^ TAMs is correlated with exhausted CD8^+^ TILs in the murine and human models of solid tumors [109]. The implementation of anti-TREM2 mAbs enhances anticancer immunity via the modulation and elimination of TAMs. The result is the stimulated infiltration of CD8^+^ TILs and the enhancement of their effector functions. It is worth noting that TREM2^+^ TAMs are especially enriched in OC patients in whom TREM2 expression is associated with the disease grade and poorer recurrence-free survival. These findings indicate that TREM2 appears as a potential target in OC immunotherapy, especially in OC patients with TAM-rich TME [109].

Moreover, Ardighieri et al. [110] have shown that, in most cases of clear cell carcinomas (CCC) that demonstrate poor prognosis and resistance to platinum-based chemotherapy, the high density of TAMs is related to poor T cell infiltration as a result of C-X-C motif chemokine ligand 10 (CXCL10) produced by M1-type macrophages. In addition, HGSOC infiltration by immune cells contains the M1 subtype of TAMs that also express TREM2 [110].

The angiogenesis factors such as angiopoietin 2 (Ang-2) and VEGF are also able to contribute to immune suppression in OC TME by repressing anticancer immune effector cells, including APCs, and to enhance the activity of Tregs, M2 type TAMs, and myeloid-derived suppressor cells (MDSCs) [111]. The implementation of antiangiogenic factors inhibits the creation of blood vessels, which plays a crucial part in cancer progression and the decrease in the level of ICPs. The result is the increasing ratio of anti- and protumoral subsets of immune cells. The proper management of antiangiogenic factors, such as bevacizumab, may be helpful in reducing immunosuppression, restoring immunity, and improving the efficiency of the ICP blockade [112]. In addition, the implementation of ICIs results in the stimulation of antiangiogenic treatment via recruiting angiomodulatory immune cells [111,113].

### 4.3. Significance of Microsatellite Instability (MSI)

Another factor that affects the response to ICIs is microsatellite instability. Microsatellites, also known as ‘short tandem repeats’, are small repetitive DNA sequences. Because of their structure, they are most likely the effect of a replication error, indicating MSI or dMMR [114]. The occurrence of dMMR marks the loss of at least one of mismatch repair (MMR)-related proteins: MutL homolog 1 (*MLH1*), MutS homolog 2 (*MSH2*), MutS homolog 6 (*MSH6*), and PMS1 homolog 2 (*PMS2*) [114,115]. Mismatch repair deficiency in cancer leads to the accumulation of genetic abnormalities and elevated tumor mutational burden (TMB) levels, as well as stimulates tumors to be highly immunogenic. These features appear to be a good foothold for ICI-based therapy [116]. Nonomura et al. [117] have found that high MSI is associated with higher CD8^+^ T cells tumor infiltration and enhanced immune response in ovarian endometrioid carcinomas [117]. The PD-1 blockade has also been proven effective and induces a permanent response in patients with either MSI-H/dMMR metastatic or unresectable non-colorectal cancer. However, only 1.6–20% of OCs are dMMR. Therefore, the implementation of ICIs does not bring the expected benefits in OC patients and is not tested on a routine basis [114,115,117,118]. Nevertheless, dihydropyrimidinase-like 2 (*DPYSL2*) and alpha kinase 2 (*ALPK2*) genes have been found to be affected by MSI frameshift events in OC and could be prospectively used to identify the occurrence of MSI [115]. Nonomoura et al. did not observe any significant correlation between MSI-H and PD-1/PD-L1 expression [117]. Sui et al. [119] have shown the association between poor response to the ICIs blocking PD-1 in MSI-H colorectal cancer and inflammation caused by neutrophils through CD80/CD86-CTLA-4 signaling in the immunosuppressive microenvironment [119].

One of the most frequently mutated oncogenes is *ARID1A* which interacts with *MSH2* and promotes MMR, while its knockdown participates in dMMR and mutation-phenotype. Shen et al. [120] have demonstrated that tumors formed by the OC cell line with *ARID1A* deficiency in syngeneic mice display elevated TIL density values, PD-L1 expression, higher mutation load, and longer OS after anti-PD-L1-antibody implementation. This suggests that *ARID1A* inhibition could enhance the effectiveness of ICIs [120].

The FDA approved pembrolizumab (anti-PD-1 mAbs) in the treatment of solid malignancies with MSI-H or MMR. The accumulation of genetic mutations in tumors results both in their recognition as non-self and in the recruitment of immune cells. Thus, ICIs appear beneficial in solid malignancies with dMMR and MSI-H. It should be highlighted that MSI-H occurs in the minority of OC cases. Thus, ICI-based monotherapy proves beneficial for a low percentage of patients. Combination therapy appears to be a promising approach [121].

### 4.4. Significance of Tumor Mutation Burden

The effectiveness of ICIs is also determined by tumor mutation burden. It is defined as the total number of DNA somatic mutations accumulated in a tumor cell. TMB is usually measured by the number of mutations per DNA megabase (Mb). A higher TMB is generally linked to an increased number of neoantigens, which may be caused by a high number of mutations. Therefore, cancer cells can be recognized more easily by the host immune system and then attacked. In general, high TMB values (≥20 mutations/Mb) are associated with a good response to ICIs in multiple tumors such as melanoma [122,123,124,125,126]. However, TMB in OC is estimated at only 1–3.5 mutations/Mb, and OC is considered to be a tumor with low TMB and expectedly low responsiveness to immunotherapy [103]. Fan et al. [127] have shown that TMB-H OC patients have higher levels of infiltrating CD8^+^ T cells, Th1 cells, Th2 cells, and Th17. Previously published studies have already associated tumor infiltration via CD8^+^ T cells with immunotherapy responsiveness [128]. Additionally, Fan et al. [127] have found a positive correlation between immune infiltration and human leukocyte antigen class II histocompatibility antigen, DO beta chain (HLA-DOB), and interferon-stimulated gene of 20 kDa protein (ISG20), on the one hand, and a negative correlation with calcium/calmodulin-dependent protein kinase IG (CAMK1G) and ubiquitin specific peptidase 51 (USP51) in OC patients [127], on the other. Nevertheless, no significant correlation between TMB and ICI response was established [98,127]. McGrail et al. [129] have analyzed data regarding over 10,000 various tumors, finding that CD8^+^ T cell infiltration in ovarian serous cystadenocarcinomas is not associated with neoantigen load and, in this group, TMB-H does not predict a beneficial impact of ICI implementation [128,129]. However, the possibility of some indirect anti-PD1 therapy response by TMB-related signature is not excluded [98,127].

### 4.5. The Regulation of ICPs by microRNA Net

MicroRNAs (miRNAs) are small molecules (20–22 nucleotides) that play a significant role in multiple biological pathways, including the regulation of immune system response and their dual activity as a tumor suppressor or oncogene activity in TME [33,118,119,120,121,122,123,130,131,132,133,134,135]. Together with transfer RNA (tRNA), ribosomal RNA (rRNA), and other regulatory RNAs, they belong to non-coding RNAs (ncRNAs) [136,137,138,139,140]. Overall, ncRNAs account for 98% of the eukaryotic genome transcript, while the remaining 2% are translated into proteins. Primary miRNAs are usually the products of RNA polymerase II transcription in the nucleus, and subsequently, they undergo multiple transformation processes to ultimately become mature miRNAs in the cytoplasm [138]. Even though miRNAs are mostly intracellular, there are also populations of circulating miRNAs and extracellular miRNAs that are displaced in the extracellular milieu, such as blood plasma or follicular fluid [139].

MiRNAs are the predominant epigenetic modulators. Their main role consists in post-transcriptional regulation and degradation of mRNA [124,129,130]. Available data suggest that miRNAs regulate almost 70% of all genes in the human genome, and their dysregulation leads to genome instability. They can also influence multiple transcripts, including the expression of oncogenes and suppressors, hence the occurrence of malignant transformation and carcinogenesis. In addition, miRNAs can control other non-coding RNAs [141,142,143,144,145,146] and play a crucial role in cellular communication, TME modification, and the promotion of cell immune escape [124,128,131,132,133,135,147].

Moreover, miRNAs are capable of modulating gene expression, in a post-transcriptional way, via ligation to the 3′-untranslated region (3′-UTR) [33,136]. It is common that each miRNA targets various transcripts, and mRNA may be targeted by a pool of miRNAs. These dependencies create a complex net of interactions [33].

Recent studies have indicated that miRNAs take part in the regulation of anti-tumor immune response. For instance, miR-200, miR-34a, and miR-513 translationally regulate the expression of PD-L1 [33,34,137,138,148,149,150]. MiRNAs also downregulate PD-L1 expression on cancer cells and CD8+ T cell infiltration, as well as reduce the angiogenesis factors in TME and increase the sensitivity to BRAF inhibitors. The combination of a BRAF inhibitor and miR-200c reportedly prevents drug resistance, boosts the host immune response against the tumor, and makes anti-tumor treatment effective at decreased dosages [151]. In various cancer types, miR-21 represents up to 10% of total miRNA. Xi et al. [152] have demonstrated that the increased percentage of miR-21-negative macrophages is associated with increased PD-L1 expression, the result of which is the inhibition of anticancer immune response [152]. Moreover, miR-28 silences PD-1, regulates cytokine secretion in cancer cells, decreases exhaustion, and improves ICI efficiency [141].

Other miRNAs, such as miR-513, miR-34a, and miR-424, take part in the regulation of PD-L1 as well. It appears that miR-424, which regulates not only PD-L1 activity but also CD80, is particularly interesting. The elevated expression of miR-424 is positively correlated with the progression-free survival (PFS) of patients with OC [33]. The decreased level of miR-424 and the increased PD-L1 expression are associated with chemoresistant phenotypes of OC tissues and cells. Moreover, PD-L1 and CD80 may be blocked by restoring the level of miR-424 via its direct ligation to 3′-UTR of their genes. The result of the PD-L1 blockade is the activation of T cells and the restoration of tumor sensitivity to chemotherapy. Thus, miR-424 is a potential factor that may enhance the OC cell’s chemo-sensitivity through the ICP blockade [118,119,120,123].

In addition, miR34a also has an impact on OC progression because it is directly transactivated by p53, which is a well-known tumor suppressor. In OC patients, *TP53* mutations are very common, especially in HGSOC (the mutation frequency is even 95%). Schmid et al. [153] have shown the inverse relationship between miR-34a expression and clinicopathological data, such as the OC type according to the Kurman and Shih classification, the overall survival rate, grading, and the status of *TP53* mutation. Moreover, the results have indicated that miR-34a exhibits an inhibitory effect on the invasion and proliferation of OC cells [63].

Guyon et al. [154] have demonstrated that the T cells exposed to anti-PD-1 agents enhance the production of exosomal miR-4315 that induces resistance to the chemotherapy-induced apoptosis in tumor cells. This phenomenon at a molecular level is related to the downregulation of proapoptotic protein Bim via exosomal miR-4315. Thus, miR-4315 could be used as a blood biomarker to detect patients that would not respond to the combination of anti-PD-1 and chemotherapy [154].

Kousar et al. [138] have classified multiple cancer-derived miRNAs that are linked to tumor evasion by upregulating PD-L1, including miR-197, miR-873, miR-16, miR-140, miR-142, miR18a, miR-138, miR34a, miR-195, miR-3609, mi-193a-3p, miR-200, miR-93, miR-15a, miR-383, miR-340, miR-17-5p, miR-93, and miR106b. Other sources also mention miR-570 and miR-513 as particles involved in the PD-L1 expression regulation [155]. The authors indicate that miRNAs participate in the processes either by binding to the 3′ UTR of PD-L1 or by targeting programmed cell death 4 (PDCD4) via the phosphoinositide 3-kinase/protein kinase B (PI3K/Akt) pathway [138,155]. Although their impact on ICI effectiveness has not been investigated in OC yet, further analysis could provide new insights that might allow a more profound understanding of the miRNA impact on OC immunotherapy. Interestingly, miR-15a and miR-15b in neuroblastoma, and miR-140 in osteosarcoma, display certain tumor suppressive properties which are gained by the PD-L1 signaling involvement [126,143,144,156,157].

The exosomal miRNAs derived from cancer cells also play a regulatory role in TME. Their immunosuppressive properties result from their capability to induce the polarization of M1-type to M2-type macrophages. Moreover, they diminish the differentiation of T helper cells via their interaction with dendritic cells [138]. The impact of miRNAs in inducing resistance to therapy was closely analyzed in previous years in relation to chemotherapy. However, the available literature data allows us to bring attention to these molecules also in regard to immunotherapy and their prospective use to improve cancer treatment [158,159].

**Figure 3:**
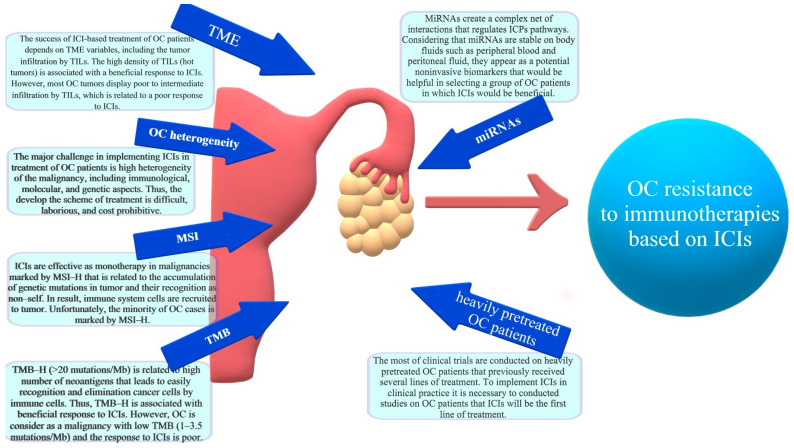
The factors triggering the OC resistance to ICI-based immunotherapy.

Considering that miRNAs are highly stable in the cytoplasm and multifarious types of body fluids, such as peritoneal fluid and blood, they can be potentially used in early cancer diagnosis and in predictions of response to implemented treatment [118,146,147,148,149]. Circulating cell-free miRNAs are the source of tumor-derived data and appear to be a useful biomarker that may help identify the premalignant stages of the disease, support OC diagnosis at the early stages, and select the group of patients in which the implementation of ICIs would be beneficial. The identification of miRNAs which are involved in the progression of OC and which regulate the ICP pathways provides a novel insight into molecular mechanisms underlying ICI resistance. Considering the influence of miRNAs on ICPs, they are potentially noninvasive biomarkers to be used for selecting the proper group of OC patients in which immunotherapies based on ICIs will prove beneficial [153,160,161,162,163,164]. Consequently, the non-responders would be detected early enough to replace the time- and cost-intensive therapy with more efficient treatment options [150,151]. The factors triggering the OC resistance to ICI-based immunotherapy are presented in Figure 3.

## 5. Hyperprogression

Some patients with solid tumors experience rapid and/or complete responses to ICIs within 12 weeks. There is also a group of patients that experience delayed response, even after 36 weeks [165,166]. The response to implementing ICIs in clinical practice has resulted in the occurrence of heterogenic and unconventional response patterns, such as hyper- and pseudoprogression [37,153].

Hyperprogression is the acceleration of tumor development as a side effect of immunotherapy [19,154]. The phenomenon was described for the first time by Chubachi et al. in a 54-year-old patient with stage IIB NSCLC treated with nivolumab. Six weeks after the administration of nivolumab (in three cycles), abrupt tumor progression was observed. Moreover, in addition to the growth of the primary tumor, multiple new nodules on the patient’s lungs and brain metastases were observed [167]. A hyperprogressive disease (HPD) may occur in various types of malignancies, including OC [19]. It should be stressed that patients with HPD have worse OS than patients experiencing standard progression [168,169,170]. The HPD incidence is reported to range from 4–29% [171,172,173,174]. A retrospective study conducted on OC patients (*n* = 89) who had received ICIs as part of clinical trials showed that 51.6% of the participants (*n* = 46) experienced HPD. As a result, ICI-based treatment was discontinued after ≤12 weeks based on the patients’ clinical or radiographic disease progression [175]. The biological mechanisms underlying hyperprogression, including senescent CD4^+^ T cells, mouse double minute 2 (MDM2), mouse double minute 4 (MDM4), and epidermal growth factor receptor (EGFR) amplification, as well as the antigen-binding Fc fragment (FcAb) regions, still remain unclear [176,177,178].

The premises concerning HPD risk factors are ambiguous. In their study, Champiat et al. showed that the age of >65 years was a risk factor for HPD [179]. However, this finding was not confirmed in other studies [158,160,161]. Moreover, Kanjanapan et al. [24] demonstrated an increased hyperprogression rate among women [24]. The studies by Kim et al. [169] and Kanjanapan et al. [180] showed that the increased number of metastasis sites was positively correlated with HPD. To date, no strong predictive factors for HPD have been identified. Notwithstanding the above, ICI-based treatment should not be limited to cancer patients based on the described factors because of the low level of proof, and the group of patients displaying HPD risk factors should be rigorously monitored to promptly identify hyperprogression [37].

## 6. Pseudoprogression

The phenomenon of initial progression followed by an objective response to the same kind of treatment is called pseudoprogression [37]. It manifests itself as an increase in tumor burden or the occurrence of new lesions that are caused by inflammation deriving from an initial response of the immune system and T cell recruitment to the tumor site as a reaction to immunotherapy based on ICPs. As a result, the tumor size is falsely increased as effector immune cells exhibit their anticancer activity [165].

However, the mechanism of pseudoprogression still remains unclear [165]. Pseudoprogression was described for the first time in melanoma after ipilimumab treatment implementation [168,169,181] and then after anti-PD-1 mAbs application (nivolumab, pembrolizumab) [182].

Further investigations have demonstrated that this phenomenon also occurs in other types of malignancies. The pseudoprogression rates vary by cancer type [165]. However, they rarely exceed 10%, e.g., in NSCLC (4–7%) and renal carcinoma (9–15%) [183].

Li et al. [184] described the case of a 47-year-old OC patient. Based on the immunohistochemistry test results that showed 10% of tumor cells expressing PD-L1, the patient received nivolumab (100mg/2 weeks). After two months, the tumor size was found to increase in computer tomography (CT). Moreover, based on the elevated levels of aspartate aminotransferase (AST) and alanine aminotransferase (ALT), the patient was diagnosed with immune-related hepatitis. The occurrence of the irAEs related to the liver suggests that the implemented ICI-based treatment is beneficial for the patient despite the enlarged tumor size. The decreased concentration of CA-125 was also observed (103.1 vs. 50.2 U/mL). Considering the clinical outcome of the patient, pseudoprogression was taken into account, and the treatment was continued. After four months, the size of the tumor decreased (50.7%), and an improvement in the patient’s outcome was observed [184].

In contrast, Passler et al. [185] demonstrated the case of a 47-year-old woman with recurrent OC. Nivolumab was implemented every three weeks in four cycles (3.0 mg/kg). The lymph node in the left groin with metastasis was twice the size compared with the values that the classic progression would suggest. However, there was no other evidence for standard progression. A stable level of CA-125 and local inflammation indicated pseudoprogression. Thus, the treatment was continued, and the size of the described lymph node with metastasis decreased, which also suggested pseudoprogression. After six treatment cycles, rectal bleeding occurred in the patient. Moreover, based on new tumor lesions occurring in the rectum, the progressive disease type was diagnosed. It should be highlighted that the size of the tumor might increase in both standard and pseudoprogression. However, the occurrence of new lesions and the infiltration of other tissues is related only to standard progression [185].

The described cases are insufficient to establish predictive or diversifying factors. Investigations should be conducted on larger OC patient groups to account for the heterogeneity of the disease. However, the cited studies determine the direction of future research.

In contrast to hyperprogression, the initial disease development in the course of pseudoprogression is followed by a positive response to ICIs. In a group of patients with pseudoprogression, the treatment should be continued. It should be highlighted that the OS of patients with pseudoprogression is improved in comparison with standard progression. To date, no clinical factors and features (i.e., CA-125, CEA, lactate dehydrogenase (LDH), age, gender) have been determined to distinguish between pseudo- and standard progression [165].

Unfortunately, there is no approach to selecting the group of patients in which pseudoprogression may occur. Selected biomarkers (i.e., cell-free DNA (cfDNA), X-ray repair cross-complementing gene (*XRCC1*), Ki67 expression, interferon regulatory factor (IRF9), small extracellular vesicles, O6-methylguanine-DNA methyltransferase methylation (MGMT), isocitrate dehydrogenase 1 (IDH1), and IL-8) and imaging approaches are helpful in the distinction of true progression from pseudoprogression. However, they are controversial and insufficient to implement in clinical practice. Presently, pseudoprogression is identified via retrospective imaging data, which results in premature discontinuation of efficient therapy. Although biopsy is an efficient diagnostic tool that is used before retrospective imaging analysis, it is an invasive method [186].

It is necessary to identify the mechanisms underlying hyper- and pseudoprogression, along with their predictive factors, to improve the implementation of treatment offered to cancer patients and to decide in which cases ICI-based treatment should be discontinued at an early stage. The need to differentiate between these two phenomena is predominant in the management of ICI-based treatment [37,187]. The identification of OC patients in whom HPD may occur is particularly crucial to preventing the rapid progression of the disease [19].

## 7. Future Directions

### 7.1. Double and Triple ICP Blockade

Similar to other malignancies, there is evidence that the implementation of a dual or triple immune checkpoint blockade may be beneficial for OC patients and may help overcome immunoresistance [5,173,174].

Although T cell activity is mostly controlled by the PD-1/PD-L1/PD-L2 pathway, other co-expressed ICPs, such as LAG-3, TIM-3, TIGIT, and DNAX accessory molecule-1 (DNAM-1; CD226), also regulate T cell activity, whether directly or indirectly [188,189,190,191].

It is well established that anti-PD-1 mAbs synergize with anti-CTLA-4 agents to fully restore T cell activity [192,193,194]. The synergistic effect is displayed as a priming and expansion of tumor-specific T cells in TME. Moreover, the dual blockade of these ICPs is a strategy to inhibit the promotion of another inhibitory axis while only one co-inhibitory molecule is blocked [192]. In spite of the synergistic effect of the dual blockade of PD-1 and CTLA-4, their simultaneous implementation leads to an increased rate of irAEs in comparison with single-agent application [178,181,182,195,196].

The TIGIT receptor is a negative regulator of T cells and NK cells [197,198,199,200]. Similar to PD-1, TIGIT is considered to be an exhaustion marker of CD8^+^ T cells. The receptor is able to regulate the anti-tumor response through CD4^+^ Tregs that are associated with tumor burden in OC patients [201]. In their preclinical studies conducted on a murine model, Chen et al. [202] demonstrated that TIGIT expression is increased in immune cells, such as Tregs. The blockade of TIGIT in mice with OC results in their beneficial survival rates as a result of Treg activity inhibition. These findings indicate that TIGIT is able to stimulate the Treg activity and plays a significant role in the creation of immunosuppression signatures in OC TME [202].

It has been shown that the TIGIT/CD155/DNAM-1 axis synergizes with the PD-1/PD-L1/PD-L2 pathway [189,190,191]. The double blockade of both pathways stimulates the effector activity of CD8^+^ T cells [202,203,204]. Banta et al. [201] have shown that costimulatory receptor DNAM-1 is a common factor of both these pathways. Both TIGIT and PD-1 are able to suppress the activity of DNAM-1, so the dual blockade is intrinsic to restoring the costimulatory signaling of DNAM-1. Thus, the single blockade of PD-1 or TIGIT via mAbs is insufficient to restore DNAM-1 functions. Hoogstad-van Evert et al. [205] have demonstrated that the OC patients with decreased DNAM-1 expression on NK cells derived from ascites have shorter survival times in comparison to the OC patients with increased DNAM-1 expression. The complex activity of the TIGIT/CD155/DNAM-1 axis, its synergistic mode of action with the PD-1/PD-L1/PD-L2 pathway, and the clinical trials on OC were described in detail in our previous paper [5].

Ongoing clinical trials also focus on the implementation of a triple ICPs blockade. The phase 1/2 study (NCT05187338) involves the combination of three mAbs, i.e., anti-PD-1 (pembrolizumab), anti-PD-L1 (durvalumab), and anti-CTLA-4 (ipilimumab), in the treatment of advanced solid tumors, including OC. The aim of the study is to establish the efficacy, safety, and survival benefits for cancer patients. The results have not been published yet [206]. Anderson et al. [207] have demonstrated, using a murine model of OC, that the triple ICP blockade (anti-PD-1, anti-TIM-3, and anti-LAG-3 mAbs) is more efficient than anti-PD-1 mAbs in monotherapy. The interactions of inhibitory receptors or ligands in TME lead to the impairment of the effector functions of T cells. This suggests that cancer cells can evade immune response via upregulating PD-L1 and ligands for LAG-3 and TIM-3. In the murine model of advanced OC, the implementation of triple ICIs results in a significant improvement of outcomes and the activity of transferred engineered T cells in comparison with the lack of significant effect after single blockade implementation [207]. Considering the complexity of a combinatory ICIs blockade, this kind of treatment may pose the risk of secondary events, including irAEs. Thus, it is important to find a suitable combination of ICIs for OC treatment. The main challenge is to develop an efficient treatment strategy without increasing the risk of irAEs occurrence [208,209].

### 7.2. Vaccines

Both vaccines and ICIs are aimed at fighting the disease via the modulation of host immune response mechanisms [194,195]. A cancer vaccine is usually understood as a vaccine against tumor-associated antigens with the addition of adjuvants activating DCs or DCs in general [210,211,212]. The first report on the potential OC vaccine development dates back to 2013 and describes a dendritic cell vaccine pulsed with autologous hypochlorous acid-oxidized OC lysate. The study showed some promising results in both mice and human preclinical experiments and prompted an attempt to adapt it to clinical practice with favorable outcomes [213]. Subsequent studies expanded the scope of the study and reported positive effects of a whole-tumor lysate-pulsed dendritic cell vaccine (OCDC) combined with bevacizumab (VEGFi) and cyclophosphamide elicited neoantigen-specific T cells on the OS rates in OC patients. Then, new evidence showed that the addition of acetylsalicylic acid (ASA) and low-dose IL-2 to OCDC, bevacizumab, and cyclophosphamide positively correlated with prolonged OS and time to progression rates [214,215]. Since numerous trials have proven the safety and potential benefits of DC vaccines, these agents could positively contribute to OC treatment outcomes [216]. Conversely, Martin-Lluesma et al. [217] have suggested that in addition to dendritic cell vaccines, B cells and macrophages could become the next agents playing a crucial role in the development of novel anti-cancer vaccines [217]. Moreover, according to Brentville et al. [218], the Modi-1 peptide vaccine consisting of a combination of citrullinated vimentin and enolase peptides could be an effective vaccine in OC patients [218].

Since FRα expression is almost exclusive for cancer tissue, and its epitopes have the capability to enhance T cell response in OC, the idea of vaccine development became reasonable and potentially achievable [219]. An attempt was made to determine whether the use of a multi-epitope anti-folate receptor vaccine (TPIV200) combined with durvalumab, a PD-L1 antibody, could improve the immunotherapy outcomes and help overcome ICI resistance. The TPIV200 vaccine consists of five highly antigenic human leukocyte antigen (HLA) peptides from FRα that are immunogenic and can evoke T cell response. The study results published by Zamarin et al. [219] in 2020 revealed that, despite the fact that vaccine-specific T cells had been produced, they were not effective enough to induce an anti-tumor response. According to the results of the phase 2 trial, there was no correlation between the response level and the antigens and PFS or OS. Therefore, the study was discontinued after phase 1 accrual. Nevertheless, the authors strongly suggest that this vaccination could potentially positively influence treatment outcomes [219].

### 7.3. Machine Learning as a Hope for Ovarian Cancer Patients

Machine learning is a subfield of artificial intelligence (AI) that succeeded in arousing interest in versatile scientific fields, including medicine. Machine learning is based on algorithms and statistical models that give computers the capability to learn and later recognize and analyze data patterns and relationships to make decisions, predictions, and recommendations based on previously unknown data [220,221,222]. There are various learning methods and models comprised in the term machine learning. In the following chapter, we will analyze its potential application in OC diagnostics. AI could provide support not only in respect of the early detection of OC but it could also help specify the genetic properties of OC [189,190,191,192].

A deep convolutional neural network (DCNN) is a machine learning algorithm that can be used for tasks such as image recognition. It learns from uploaded data during training and then makes predictions on previously unseen data. A DCNN model could potentially be suitable for distinguishing between benign and malignant adnexal tumors based on ultrasound images. The technique is capable of interpreting the nature of the ultrasound scans provided using an algorithm originating from numerous previous scan analyses and diagnoses. This tool was developed by Gao et al. [223] and is based on retrospective images of adnexal masses from multiple healthcare centers in China. The DCNN-assisted tumor evaluation has displayed certain advantages in terms of the distinction between subtle image details and features, easily overlooked by the human eye, along with better efficiency, a versatile database used in algorithm development, and smooth distribution in less experienced healthcare centers. This tool could also be used by medical professionals as a support in their real-time ultrasound examination in clinical practice. However, there are several factors that add up to malignancy risk evaluation, such as genomic characteristics, BRCA mutation status, or histological subtype. Since molecular testing in many cases is not easily accessible, the authors have emphasized that further investigation and development of DCNN could help determine the OC subtype only with an AI-based application [223].

Another study has demonstrated the application of machine learning models and statistics in the classification models aimed at developing efficient blood biomarkers for the early diagnosis of OC [224]. The database contains laboratory blood results consisting of three subgroups: routine blood count (1), general blood chemistry (2), and tumor markers (3), including carbohydrate antigen 72-4 (CA72-4), alpha-fetoprotein (AFP), carbohydrate antigen 19-9 (CA19-9), CA-125, carcinoembryonic antigen (CEA), human epididymis protein 4 (HE4), and clinical features such as menopause status and age. The authors used various machine learning tools, including Random Forest (RF), Gradient Boosting Machines (GBM), and light gradient boosting machines (LGBM) that, when combined with statistical tests, were capable of processing the datasets provided in terms of significant feature finding, feature association finding, and OC prediction [224]. This low-cost diagnostic tool could be a quality assistance for physicians, shortening the entire diagnostic process. The accuracy attributed to this method of malignant-or-benign differentiation is estimated at over 90% [224].

Another application of machine learning refers to second-harmonic generation (SHG) imaging that provides a quick and non-invasive method of OC diagnosis. More specifically, SHG provides a visualization of tissue structures, including collagen. Collagen remodeling is linked to OC carcinogenesis and progression, and the characteristics of collagen fibers vary depending on ovarian tissue origin. When combined with a machine learning model, SHG is useful in distinguishing borderline tumors from malignant and benign ones [225].

Machine learning algorithms and bioinformatics can also be used to analyze multiple large gene datasets to identify and validate genes with a potential diagnostic value. Liu et al. [226] have focused on OC genome exploration based on Gene Expression Omnibus (GEO), the Cancer Genome Atlas (TCGA), and the Genotype-Tissue Expression (GTEx) cohort datasets, with the application of machine learning algorithms. Moreover, the authors have investigated the function and pathways that are involved in the interdependence between those characteristics, diagnosis-related genes, and immune cell infiltration in OC to be analyzed at a later stage [226].

Firstly, they developed a tool to provide insight and detect differentially expressed genes (DEGs) in OC and non-OC tissues. Pieces of information about the selected genes relevant for OC were supplemented by numerous datasets to eventually undergo validation in terms of diagnostic relevance. Additionally, the authors investigated whether DEGs and immune cell infiltration could be related. According to the study results, out of 590 identified DEGs, 10 genes, i.e., budding uninhibited by benzimidazoles 1 (BUB1), adenosine 5′-triphosphate–binding cassette subfamily B member 1 (ABCB1), secreted frizzled-related protein 1 (SFRP1), innate immunity activator (INAVA), transmembrane protein 139 (TMEM139), mitotic checkpoint serine/threonine-protein kinase BUB1 beta (BUB1B), phosphoserine aminotransferase 1 (PSAT1), phosphodiesterase 8B (PDE8B), folate receptor alpha (FOLR1), and homeobox A13 (HOXA13), are involved in biological cell functions and could affect immune infiltration levels in OC [226].

Numerous attempts to directly apply machine learning in ICI response prediction have already been made. Even though OC-specific algorithms are yet to be developed, there are some promising study results in respect of other cancer types, including melanoma, glioblastoma, and hepatocellular carcinoma [227,228,229,230,231,232]. Johannet et al. [227] have created a DCNN classifying whole-slide images to predict which melanoma patients would more likely benefit from ICIs or progress during the therapy, and the nuclei characteristics were found to be crucial in the construction of algorithms [227]. In 2019, Harder et al. [229] presented a DCNN model that successfully predicted ipilimumab response in malignant melanoma. Their model used whole-slide images of different materials, such as lymph nodes and skin, to identify their cells with emphasis on immune cell densities and distances between them. The superior model developed in the process turned out to be a decision tree and included the concepts of distribution and density of CD8^+^ and CD3^+^ in TME. The study revealed that a high ratio of intratumoral CD8^+^ infiltration to CD8^+^ and CD3^+^ densities in surrounding tissues indicated a good therapy response [229].

Another machine learning model to predict ICI response was created by Zhang et al. [230] for glioblastoma, and their method analyzed the tumor-infiltrating immune cell-associated long noncoding ribonucleic acids (TIIClnc) signature using purified immune cells, glioblastoma cell lines and glioblastoma tissues transcriptome data. The developed TIIClnc signature was a marker of immune infiltration correlated with CD8^+^, PD-1, and PD-L1 [230]. A paper by Wang et al. [231], dated 2020, was the starting point for examining the role of cancer stem cells in tumorigenesis and resistance to therapy in glioblastoma. The authors performed an integrated multiomic analysis using the Tumor Immune Dysfunction and Exclusion (TIDE) algorithm to study the correlation between stemness and immuno- and chemotherapy response in glioblastoma patients. Based on their findings, they established a novel stemness classification that helped them identify which patients would more likely benefit from ICIs [231]. With a view to predicting ICI effectiveness in hepatocellular carcinoma, Chen et al. [232] created cancer-stem-cell-related clusters using machine learning algorithms that combined datasets of genome information. After that, it was possible to categorize patients according to stemness subtypes, which were found to be strongly related to immune infiltration modulation and able to predict their immunogenomic expressions, tumor immune microenvironment status, and thus immunotherapy susceptibility [232].

Recently, Kong et al. have built a machine learning framework that could make an accurate prognosis of ICI-based treatment effectiveness, relying on network-based biomarkers. Moreover, according to the results established for ICI treatment outcomes in melanoma, metastatic gastric cancer, and bladder cancer, the authors have found that predictions made using network-based biomarkers are more precise than those based on the expression levels of ICI targets, including PD1, PD-L1, or CTLA-4 [228].

## 8. Conclusions

Given the limited efficacy of the current treatment options for OC patients, novel therapeutic approaches are urgently required. The immunotherapies based on ICIs have turned out to be game-changers in the treatment of cancer types with poor prognoses, such as melanoma. Thus, this kind of therapy appears to be a promising approach to breaking immunosuppression in OC TME. Unfortunately, the preclinical studies and clinical trials conducted to date have shown that OC tumors are non-inflamed, and the response to ICIs among OC patients is insufficient, especially if monotherapy is applied. Thus, the combination of ICIs with other biological drugs, such as PARPi or antiangiogenic factors (VEGFi), aimed at sensitizing the tumor to this kind of treatment seems to be a promising approach. Moreover, it is crucial to examine various combinations of ICIs, also in double and triple blockades, to break the immunosuppression in OC TME and to overcome immunoresistance.

Considering that the majority of studies are conducted on recurrent OC patients that previously received several treatment lines, further investigation of their efficiency as first-line treatment is highly needed to break the ICI immunosuppression. Another challenge is posed by the proper selection of OC patients and the development of predictive biomarkers that would help identify the OC individuals in whom this treatment would prove beneficial. The understanding of the mechanisms underlying immunoresistance, including immunological, genetic, and molecular aspects, is crucial to developing efficient immunotherapy for OC patients and improving their clinical outcomes.

## Figures and Tables

**Table 1:** The advanced-phase clinical trials (phases 3 and 4) concerning ICIs in OC treatment.

NCT Number	Acronym	Condition	mAbs Anti-ICPs	Additional Drugs	Participants	Phase	Company	Ref.
NCT03598270	ANITA	Recurrent ovarian carcinoma	Atezolizumab	placebo carboplatin paclitaxel niraparib gemcitabine PLD	414	3	Grupo Español de Investigación en Cáncer de Ovario	[74]
NCT03522246	ATHENA	Epithelial ovarian cancer	Nivolumab	rucaparib placebo oral tablet placebo IV infusion	1000	3	Clovis Oncology, Inc.	[75]
NCT03740165	-	Epithelial ovarian cancer	Pembrolizumab	placebo for pembrolizumab carboplatin paclitaxel olaparib placebo for olaparib bevacizumab docetaxel	1367	3	Merck Sharp & Dohme LLC	[76]
NCT03602859	FIRST	First-line treatment of stage III/IV non-mucinous epithelial OC	Dostarlimab (TSR-042)	niraparib standard care dostarlimab-placebo niraparib-placebo	1403	3	Tesaro, Inc.	[77]
NCT05116189	-	Platinum-resistant recurrent ovarian cancer	Pembrolizumab	paclitaxel bevacizumab placebo for pembrolizumab docetaxel	616	3	Merck Sharp & Dohme LLC	[78]
NCT03353831	-	early relapse ovarian cancer	Atezolizumab	bevacizumab chemotherapy placebos	550	3	AGO Research GmbH	[79]
NCT02580058	JAVELIN OVARIAN 200	Platinum resistant/ refractory ovarian cancer	Avelumab	PLD	566	3	Pfizer	[80]
NCT03642132	JAVELIN OVARIAN PARP -	Untreated advanced ovarian cancer	Avelumab	chemotherapy + avelumab followed by avelumab + talazoparib chemotherapy + bevacizumab followed by bevacizumab chemotherapy, followed by talazoparib maintenance	79	3	Pfizer	[81]
NCT02718417	JAVELIN OVARIAN 100	Previously untreated patients with epithelial ovarian cancer	Avelumab	carboplatin paclitaxel	998	3	Pfizer	[82]
NCT03038100	IMagyn050	Newly-diagnosed stage III or stage IV ovarian cancer	Atezolizumab	paclitaxel carboplatin bevacizumab atezolizumab placebo	1301	3	Hoffmann-La Roche	[83]
NCT02891824	ARCAGY/GINECO GROUP	Late relapse ovarian cancer	Atezolizumab	atezolizumab + avastin + platinum-based chemotherapy placebo + avastin + platinum-based chemotherapy	614	3	ARCAGY/GINECO GROUP	[84]
NCT02839707	-	Recurrent ovarian cancer	Atezolizumab	bevacizumab computed tomography PLD hydrochloride quality-of-life assessment	444	2/3	National Cancer Institute (NCI)	[85]
NCT03755739	-	Ovarian cancer	Pembrolizumab, ipilimumab	immune checkpoint inhibitors such as pembrolizumab, ipilimumab plus chemotherapy	200	2/3	Second Affiliated Hospital of Guangzhou Medical University	[86]
NCT03651206	ROCSAN	Recurrent ovarian carcinosarcoma	Dostarlimab	niraparib niraparib + dostarlimab chemotherapy drugs	196	2/3	ARCAGY/GINECO GROUP	[87]
NCT04679064	NItCHE-MITO33	Recurrent ovarian cancer patients not a candidate for platinum retreatment	Dostarlimab	niraparib pegylated liposomal doxorubicin paclitaxel gemcitabine topotecan bevacizumab	427	3	Fondazione Policlinico Universitario Agostino Gemelli IRCCS	[88]

**Table 2:** Selected early-phase clinical trials concerning ICIs in OC treatment.

NCT Number	Acronym	Condition	mAbs Anti-ICPs	Additional Drugs	Participants	Phase	Company	Ref.
NCT04611126	-	Metastatic ovarian cancer	Ipilimumab Nivolumab Relatlimab	cyclophosphamid fludarabine phosphate tumor-infiltrating lymphocytes infusion	18	1/2	Inge Marie Svane	[91]
NCT03219268	-	Ovarian cancer	Tebotelimab Margetuximab	-	353	1	MacroGenics	[90]
NCT03538028	-	Advanced ovarian cancer	INCAGN02385	-	22	1	Incyte Biosciences International Sàrl	[92]
NCT03849469	DUET-4	Advanced ovarian cancer	Xmab^®^22841 Pembrolizumab	-	78	1	Xencor, Inc.	[89]
NCT04354246	-	Advanced ovarian cancer	COM902 COM701 (antiCD112R) pembrolizumab.	-	110	1	Compugen Ltd.	[93]
NCT05026606	-	Recurrent ovarian clear cell adenocarcinoma Recurrent platinum-resistant ovarian carcinoma	Etigilimab nivolumab	-	20	2	M.D. Anderson Cancer Center	[94]

**Table 3:** Selected clinical trials concerning ICIs in monotherapy and combined therapy.

mAbs Anti-ICPs	Additional Drugs	NCT Number
Pembrolizumab	- (monotherapy)	NCT05368207 NCT04575961 NCT03732950 NCT04602377 NCT03430700 NCT04375956 NCT02644369 NCT03012620
	chemotherapy	NCT03734692 NCT05467670 NCT04387227 NCT02766582 NCT03410784 NCT03755739 NCT02520154 NCT03126812
	VEGFi + chemotherapy	NCT03596281 NCT03275506 NCT05116189
	VEGFi + PARPi + chemotherapy	NCT03740165 NCT05158062
	PARPi	NCT04417192
	VEGFi + PARPi	NCT04361370
	PY314	NCT04691375
	KVA12123	NCT05708950
	Anti-CTLA4	NCT04140526
	Modified vaccinia virus Ankara vaccine expressing p53	NCT03113487
Nivolumab	PARPi (Rucaparib)	NCT03522246
	PARPi + VEGFi	NCT02873962
	Chemotherapy + PARPi	NCT03245892
	Etigilimab	NCT05715216
	NY-ESO-1 peptide vaccine	NCT05479045
Atezolizumab	Chemotherapy + PARPi	NCT03598270
	Chemotherapy + VEGFi	NCT03353831 NCT02891824 NCT02839707
	VEGFi	NCT04510584
Durvalumab	Olaparib + Bevacizumab	NCT04015739
Durvalumab + Tremelimumab	(ICIs combination)	NCT03026062
Tremelimumab	PARPi	NCT04034927
Nivolumab + Ipilimumab	(ICIs combination)	NCT03355976 NCT03508570 NCT02498600
Ipilimumab +Pembrolizumab +Durvalumab	(ICIs combination)	NCT05187338

## Data Availability

Not applicable.

## Abbreviations

3′-UTR	3′-untranslated region
ABCB1	adenosine 5′-triphosphate–binding cassette subfamily B member 1
AFP	alpha-fetoprotein
AI	artificial intelligence
ALPK2	alpha kinase 2
ALT	alanine aminotransferase
Ang-2	angiopoietin 2
APCs	antigen-presenting cells
ARID1A	AT-rich interactive domain-containing protein 1A
ASA	acetylsalicylic acid
AST	aspartate aminotransferase
BRAF	B-Raf proto-oncogene, serine/threonine kinase
*BRCA*	breast cancer gene
BTC	biliary tract cancers
BUB1	budding uninhibited by benzimidazoles 1
BUB1B	mitotic checkpoint serine/threonine-protein kinase BUB1 beta
CA-125	cancer antigen 125
CA19-9	carbohydrate antigen 19-9
CA72-4	carbohydrate antigen 72-4
CAMK1G	calcium/calmodulin-dependent protein kinase 1G
CCC	clear cell carcinomas
CCL2	chemokine (C-C motif) ligand 2
CCL22	C-C motif chemokine 22
CD	cluster of differentiation
CEA	carcinoembryonic antigen
cfDNA	cell-free DNA
cHL	classical Hodgkin Lymphoma
CRC	colorectal cancer
cSCC	cutaneous squamous cell carcinoma
CSF-1	colony stimulating factor 1
CT	computer tomography
CTLA-4	cytotoxic T-lymphocyte-associated antigen 4
CXCL10	C-X-C motif chemokine ligand 10
DCNN	deep convolutional neural network
DCs	dendritic cells
DEG	differentially expressed gene
dMMR	deficient mismatch repair
DNAM-1	DNAX accessory molecule-1
DPYSL2	dihydropyrimidinase like 2
EGF	epidermal growth factor
EGFR	epidermal growth factor receptor
ER	estrogen receptor
ERK	extracellular signal-regulated kinase
ESMO	European Society for Medical Oncology
FcAb	antigen-binding Fc fragment
FDA	Food and Drug Administration
FIGO	International Federation of Gynecology and Obstetrics
FOLR1	folate receptor alpha
FOXM1	forkhead box M1
FRα	folate receptor alpha
GBM	Gradient Boosting Machines
GEO	Gene Expression Omnibus
GTEx	The Genotype-Tissue Expression
HCC	hepatocellular carcinoma
HE4	human epididymis protein 4
HGF	hepatocyte growth factor
HGSOC	high-grade serous ovarian carcinoma
HLA	human leukocyte antigen
HLA-DOB	histocompatibility antigen, DO beta chain
HNSCC	head and neck squamous cell carcinoma
HOXA13	homeobox A13
HPD	hyperprogressive disease
ICI	immune checkpoint inhibitor
ICP	immune checkpoint
IDH1	isocitrate dehydrogenase 1
IFN-γ	interferon γ
IL	interleukin
INAVA	innate immunity activator
irAEs	immune-related adverse events
IRF9	interferon regulatory factor 9
ISG20	interferon-stimulated gene of 20 kDa protein
JAK-STAT	Janus kinase/signal transducers and activators of transcription
KRAS	Kirsten rat sarcoma viral oncogene homolog
LAG-3	lymphocyte activation gene 3
LDH	lactate dehydrogenase
LGBM	light gradient boosting machine
mAbs	monoclonal antibodies
MAP	mitogen-activated protein
MAPK	mitogen-activated protein kinase
Mb	megabase
MCC	Merkel cell carcinoma
MDM2	mouse double minute 2
MDM4	mouse double minute 4
MDSC	myeloid-derived suppressor cells
MGMT	O6-methylguanine-DNA methyltransferase methylated
miRNA	microRNA
MLH1	MutL homolog 1
MMR	mismatch repair
MO/MA	monocytes/macrophages
MSH2	MutS homolog 2
MSH6	MutS homolog 6
MSI	microsatellite instability
MSI-H	microsatellite instability-high
MSI-H	high microsatellite instability
NCCN	National Comprehensive Cancer Network
ncRNAs	non-coding RNAs
NK cell	natural killer cell
NSCLC	non-small-cell lung cancer
OC	ovarian cancer
OCDC	whole-tumor lysate-pulsed dendritic cell vaccine
OS	overall survival
PARPi	poly(ADP-ribose) polymerase inhibitor
PD-1	Programmed cell death receptor 1
*PDCD1*	Programmed Cell Death 1
PDCD4	programmed cell death 4
PDE8B	phosphodiesterase 8B
PD-L1	Programmed death-ligand 1
PD-L2	Programmed death-ligand 2
PFS	progression-free survival
PI3K/Akt	phosphoinositide 3-kinase/protein kinase B
PIK3CA	phosphatidylinositol-4,5-bisphosphate 3-kinase catalytic subunit α
PLD	pegylated liposomal doxorubicin
PMLBCL	primary mediastinal large B-cell lymphoma
PMS2	PMS1 homolog 2
PR	progesterone receptor
PSAT1	phosphoserine aminotransferase 1
PTEN	phosphatase and tensin homolog deleted on chromosome ten
RCC	renal cell carcinoma
RF	random forest
rRNA	ribosomal RNA
SCLC	small cell lung cancer
SFRP1	secreted frizzled-related protein 1
SHG	second-harmonic generation
TAM	Tumor-associated macrophage
TCGA	The Cancer Genome Atlas
TCR	T cell receptor
TGF-β	transforming growth 276 factor β
TIDE	Tumor Immune Dysfunction and Exclusion algorithm
TIGIT	T cell immunoglobulin and ITIM domain
TIIClnc	tumor-infiltrating immune cell-associated long noncoding ribonucleic acids
TIL	tumor-infiltrating lymphocytes
TIM-3	T cell immunoglobulin, mucin domain-containing protein 3
TMB	tumor mutational burden
TMB-H	high tumor mutational burden
TME	tumor microenvironment
TMEM139	transmembrane protein 139
TNF-α	tumor necrosis factor α
TP53	Tumor protein P53
TPIV200	a multi-epitope anti-folate receptor vaccine
TREM2	triggering receptor expressed on myeloid cells 2
tRNA	transfer RNA
*UBR5*	ubiquitin 288 protein ligase E3 component n-recognin 5
USP51	ubiquitin specific peptidase
VEGF	vascular endothelial growth factor
VEGFi	vascular endothelial growth factor inhibitor
WHO	World Health Organization
*XRCC1*	X-ray repair cross-complementing gene
